# The efficacy of ampicillin compared with ceftriaxone on preventing cesarean surgical site infections: an observational prospective cohort study

**DOI:** 10.1186/s13756-018-0304-6

**Published:** 2018-01-22

**Authors:** Srisuda Assawapalanggool, Nongyao Kasatpibal, Supatra Sirichotiyakul, Rajin Arora, Watcharin Suntornlimsiri, Anucha Apisarnthanarak

**Affiliations:** 1 Infection Control Section, Mae Sot Hospital, 175/16 Sri Panich Road, Mae Sot Sub-district, Mae Sot District, Tak, 63110 Thailand; 2 Division of Nursing Science, Faculty of Nursing, Chiang Mai University, 110 Inthawaroros Road, Sriphum Sub-district, Muang District, Chiang Mai, 50200 Thailand; 3 Department of Obstetrics and Gynecology, Chiang Mai University, 110 Inthawaroros Road, Sriphum Sub-district, Muang District, Chiang Mai, 50200 Thailand; 4 Department of Obstetrics and Gynecology, Lampang Hospital, 280 Phaholyothin Road, Hua Wiang Sub-district, Muang District, Lampang, 52000 Thailand; 5 Department of Obstetrics and Gynecology, Nakornping Hospital, 159 Chotana Road, Don Kaew Sub-district, Mae Rim District, Chiang Mai, 50180 Thailand; 6 Division of Infectious Diseases, Thammasart University Hospital, 95 Moo 8 Paholyotin Road, Klongluang District, Pathum Thani, 12120, Thailand

**Keywords:** ampicillin, ceftriaxone, propensity score, observational study, cesarean infections

## Abstract

**Background:**

Cesarean surgical site infections (SSIs) can be prevented by proper preoperative antibiotic prophylaxis. Differences in antibiotic selection in clinical practice exist according to obstetricians’ preferences despite clear guidelines on preoperative antibiotic prophylaxis. This study aimed to compare the efficacy of ampicillin and ceftriaxone in preventing cesarean SSIs.

**Methods:**

The observational prospective cohort study was conducted at a tertiary hospital in Thailand from 1 January 2007 to 31 December 2012. Propensity scores for ceftriaxone prophylaxis were calculated from potential influencing confounders. The cesarean SSI rates of the ceftriaxone group vs. those of the ampicillin prophylactic group were estimated by multilevel mixed-effects Poisson regression nested by propensity score.

**Results:**

Data of 4149 cesarean patients were collected. Among these, 911 patients received ceftriaxone whereas 3238 patients received ampicillin as preoperative antibiotic prophylaxis. The incidence of incisional SSIs was (0.1% vs. 1.2%; *p* = 0.001) and organ space SSIs was (1.2% vs. 2.9%; *p* = 0.003) in the ceftriaxone group compared with the ampicillin group. After adjusting for confounders, the rate ratios of incisional and organ/space SSIs in the ceftriaxone compared with the ampicillin group did not differ (RR, 0.23; 95% CI 0.03–1.78), and (RR, 1.62; 95% CI 0.83–3.18), respectively.

**Conclusion:**

These data indicate no difference exists between ampicillin and ceftriaxone to prevent SSIs after cesarean section. Ampicillin may be used as antibiotic prophylaxis in cesarean section.

**Electronic supplementary material:**

The online version of this article (10.1186/s13756-018-0304-6) contains supplementary material, which is available to authorized users.

## Background

Cesarean section is an operation that has been increasingly performed in the USA [1] and also in the Asian region [2]. Surgical site infections (SSIs) occurring after this operation were estimated at 1.5 to 3.8% in the USA [3] and 0.9% in Thailand [4]. Proper surgical antibiotic prophylaxis is part of the SSI prevention bundles that have been recommended to surgical patients [5] including all cesarean patients [6, 7] to reduce the rate of post-cesarean SSIs [8]. It should be administered within 60 min before incision to ensure adequate concentration in the blood and tissue throughout the operation [7, 9].

Intraoperative redosing is required whenever the elapsed time from the first dose of antibiotic prophylaxis and operative time is longer than two half-lives of the antibiotic [9] because the failure of redosing during a long operation increases the risk of SSIs [10]. Redosing during surgery is also needed in the case of massive perioperative blood loss [9]. Increasing dosage may be required for obese patients to achieve the optimal level in the tissues [11]. Currently, a single 1-g intravenous dose of cefazolin [12] or at a higher dose of 2-g [9] is recommended as the first-line preoperative antibiotic of choice for cesarean patients. In spite of clear guidelines on preoperative antibiotic prophylaxis, differences in clinical practices remain, depending on obstetricians’ preference. Ceftriaxone and ampicillin have been prescribed in our setting and some others in Thailand.

Only one randomized controlled trial has been conducted to directly compare the efficacy of ampicillin vs. ceftriaxone concerning cesarean SSIs prevention and it lacked statistical power [13]. We therefore, conducted this study to compare the efficacy of ampicillin and ceftriaxone in the real context of preventing cesarean SSIs.

## Methods

### Study design and setting

This observational prospective cohort study was conducted from January 2007 to December 2012 in a tertiary center located on the northwest Thai-Myanmar border, where an average of 800 cesarean cases are performed annually.

### Patients

Data of women undergoing cesarean operations during the study period were collected from our local infection control database. All patients undergoing cesarean operations included in the study received either ampicillin or ceftriaxone as the preoperative antibiotic prophylaxis accordingly to surgeon’s preference. In our setting, both ampicillin and ceftriaxone were 2-g dosages. Both preoperative antibiotic prophylaxis regimens were administered at the time of cord-clamp or within 60 min before incision and were discontinued for no longer than 24-h postoperatively. In total, all patients received either 3 doses of ampicillin or 2 doses of ceftriaxone according to standing order protocol in this setting. Patients with chorioamnionitis, prolonged ruptured membranes > 18 h, cases that switched to other regimens during the perioperative period or received other regimens and cases with improper administration time (> 60 min before incision) were excluded from the study. All six obstetricians in our setting followed the Centers for Disease Control and Prevention (CDC) guidelines of SSI prevention [14] and also prescribed preoperative antibiotic prophylaxis for all patients undergoing cesarean operation during the study period.

### Data collection

Demographic data, background medical problems, antenatal care history and referral data were collected. Perinatal and perioperative data, e.g., chief complaints, clinical findings on physical examination, progression of labor, transvaginal procedures before undergoing operation, surgeons, place of antenatal care, PROM, being referred from local settings, failure to induce labor, being preterm, having an emergency operation, being affected by acquired human immunodeficiency syndrome, multiple gestations, wound class and operative details, were also gathered. Patients in our center had not been screened for group B *Streptococcus*. Clinical outcomes and data from postdischarge surveillance system were provided 30-day follow-up and recorded. Wounds of all patients were checked on the third postoperative day, whenever necessary and on the day of discharge. One week and 30-day follow-up was appointed for every patient. Any patient with wound complications or gynecologic problems was referred for consultation at our center. Loss of attending any follow-up appointments in our network was classified as lost to follow-up. This observational study was approved by the Research Ethics Committee 4, Faculty of Medicine, Chiang Mai University (reference no. 186/2013).

### Clinical response, clinical outcome and definitions

The outcome of this study was cesarean SSIs incidence of both antibiotic cohorts and was defined according to the National Healthcare Safety Network surveillance of CDC criteria [15]. Case surveillance was conducted by infection control nurses and personnel in the postdischarge surveillance system according to the surveillance criteria.

### Statistical analysis

Continuous data were described and properly compared using student t-test or Wilcoxon rank-sum test depending on their distributions. Fisher’s exact test was used to compare discrete data.

Propensity scores for ceftriaxone prophylaxis were calculated from potential factors influencing or placing patients more likely in one preoperative antibiotic group than another. These included individual surgeons, private antenatal care, premature rupture of the membranes (ROM), being referred from local settings, failure to induce labor, being preterm, having an emergency operation, affected by acquired human immunodeficiency syndrome, multiple gestations and wound class ≥3.

The cesarean SSI rates of ceftriaxone vs. those of the ampicillin prophylactic group were estimated by multilevel mixed-effects Poisson regression and were expressed as rate ratio (RR). The potential confounding factors revealed from a related study [16] including ethnic minority, anemia, being referred, preterm, primigravida, pelvic examination ≥5 times before cesarean operation, foul-smelling amniotic fluid, wound class ≥3, operation time > 55 min, and emergency operation were all adjusted in the fixed-effects part of the model nested within propensity scores as random-effects part to fully control the effect of confounding bias and residual confounders because these factors could not be constructed homogenous subclass in propensity score [17]. We found some confounders by indication were also risk factors for cesarean SSIs in our setting [16] including being primigravida, preterm, being referred, wound class ≥3, emergency operation and multiple gestations. We therefore included these confounders in multivariable multilevel regression model because these potential confounding factors could distort the outcome. In addition, double-adjustment could reduce residual confounding biases [18, 19] and another reason was that all confounders should be fully controlled in the efficacy study [20]. We used Kaplan-Meier survival curves to compare survival of caesarean SSIs between the ceftriaxone and ampicillin groups. *P*-values were obtained from Cox’s survival regression analysis. We considered P-values of less than 0.05 as statistically significant. All data were analyzed using STATA®, version 11.2 (StataCorp).

## Results

**Fig. 1:**
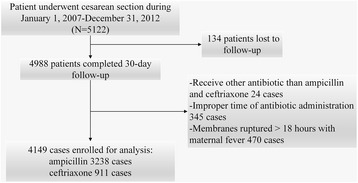
Data flow of the study

In total, 5122 patients underwent cesarean sections. Of these, 134 patients were lost to follow-up. Of the remaining patients, 24 received antibiotics other than ampicillin and ceftriaxone as preoperative prophylaxis, 345 patients received improper antibiotic-administration time and 470 patients presented ROM > 18 h accompanied by fever before the cohort zero-th time, and all were excluded from the study (Fig. 1). Thus, a total of 4149 patients were included in the study. Among these, 911 patients received ceftriaxone and 3238 patients received ampicillin as preoperative antibiotic prophylaxis. Baseline characteristics and propensity scores differed between the two comparison groups and the results are shown in Table 1.

**Table 1:** Characteristics of patients receiving antibiotic prophylaxis with ceftriaxone and ampicillin (*n* = 4149)

Characteristic	Ceftriaxone (*n* = 911)		Ampicillin (*n* = 3238)		*p*-value
	n	%	n	%	
Age (yr)					
< 20	24	2.6	209	6.5	
20–34	691	75.9	2391	73.8	
≥ 35	196	21.5	638	19.7	
Mean ± SD	30.2	±5.3	28.9	±6.2	< 0.001
Ethnic minority	130	14.3	1788	55.2	< 0.001
Education level					
None	88	9.7	1513	46.7	< 0.001
Primary	99	10.9	598	18.5	
Intermediate	236	25.9	673	20.8	
College/higher	488	53.5	454	14.0	
Antenatal care					
No	3	0.3	72	2.2	< 0.001
Camp	40	4.4	827	25.5	
Hospital	868	95.3	2339	72.3	
Body weight (kg)					
≥ 80	155	17.0	408	12.6	
Mean ± SD	69.0	±11.7	65.4	±12.4	< 0.001
Hb (g/dL)					
< 11	145	15.9	617	19.1	
Mean ± SD	11.5	±1.0	11.4	±1.2	0.008
HIV positive	5	0.6	124	3.8	< 0.001
Referral	48	5.3	1070	33.1	< 0.001
Gravida					
Primigravida	308	33.8	1140	35.2	
Median (IQR)	2	(1–2)	2	(1–3)	0.002
Multiple gestations	13	1.4	123	3.8	< 0.001
GA (weeks)					
< 37	26	2.9	425	13.1	
Mean ± SD	38.6	±1.3	38.4	±2.4	0.152
PV ≥5 occasions before surgery	17	1.9	405	12.5	< 0.001
Amniotic fluid					
Clear	896	98.4	2757	85.2	< 0.001
MSAF	11	1.2	468	14.4	
Foul smelling	4	0.4	13	0.4	
ROM duration (hr)					
Median (IQR)	0	(0–0)	0	(0–2.3)	< 0.001
Fetal death	5	0.6	38	1.2	0.136
ASA score ≥ 3	19	2.1	198	6.1	< 0.001
Wound class ≥3	15	1.7	364	11.2	< 0.001
Operation time (min)					
≥ 55	93	10.2	1209	37.3	
Median (IQR)	40	(35–45)	50	(40–60)	< 0.001
Emergency	214	23.5	2487	76.8	< 0.001
EBL (mL)					
≥ 500	121	13.3	1956	60.4	
Median (IQR)	300	(300–400)	500	(400–500)	< 0.001
UTI	1	0.1	9	0.3	0.701
Skin incision line					
Midline	119	13.1	1492	46.1	< 0.001
Pfannelstein	792	86.9	1746	53.9	
Addition procedures					
Appendectomy/TR	542	59.5	1432	44.2	< 0.001
Propensity score					
Mean ± SD	0.86	±0.27	0.04	±0.11	< 0.001
Median (IQR)	0.96	(0.92–0.96)	0.02	(0.01–0.03)	< 0.001

*ASA* American Society of Anesthesiology, *MSAF* Meconium stained amniotic fluid, *dL* Decilitre, *EBL* Estimated blood loss, *g* Gram, *GA* Gestational age, *Hb* Hemoglobin, *HIV* Human immunodeficiency virus, *hr.* Hours, *IQR* Interquartile range, *kg* Kilogram, *min* Minute, *PV* Pelvic examinations, *ROM* Rupture of amniotic membranes, *SD* Standard deviation, *SSIs* Surgical site infections, *TR* Tubal resection, *UTI* Urinary tract infection, *mL* Millilitre, *yr.* Years

The incidence of incisional SSIs was 0.1% (1/911) vs. 1.2% (39/3238); *P* = 0.001 and organ space SSIs was 1.2% (11/911) vs. 2.9% (94/3238); *P* = 0.003 in the ceftriaxone group compared with the ampicillin group (Table 2). Among 145 patients, who developed cesarean SSIs, 46 cases (31.7%) were investigated for pathogens. Negative finding resulted in 26 cases, positive one isolation resulted in 15 cases and two isolations resulted in 5 cases. Resistant organisms to preoperative antibiotic comprised 14 isolates in the ampicillin group and one in the ceftriaxone group. The remaining 10 isolates were susceptible to ampicillin (Table 3).

**Table 2:** Comparison of cesarean surgical site infections between patients receiving ceftriaxone vs. those receiving ampicillin

Characteristic	Ceftriaxone (*n* = 911)		Ampicillin (*n* = 3238)		*p*-value
	n	%	n	%	
Total Cesarean SSIs					
yes	12	1.3	133	4.1	< 0.001
no	899	98.7	3105	95.9	
Incisional SSIs					
yes	1	0.1	39	1.2	0.001
no	910	99.9	3199	98.8	
Organ/space SSIs					
yes	11	1.2	94	2.9	0.003
no	900	98.8	3144	97.1	

*SSIs* Surgical site infections

**Table 3:** Pathogens isolated from 51 cesarean surgical site infections

Pathogen	Number of isolates (*n* = 51)	Percentage of isolates
No growth	26	50.9
Susceptible to antibiotic used		
*Klebsiella pneumoniae*	4	7.8
*Escherichia coli*	3	5.8
*Acinetobacter baumannii*	2	3.9
*Proteus mirabilis*	1	2.0
Resistant to antibiotic used		
*Staphylococcus aureus*	5	9.8
Coagulase-negative *Staphylococci*	5	9.8
*Enterococcus faecalis*	1	2.0
*Enterococcus faecium*	1	2.0
*Escherichia coli*	1	2.0
*Pseudomonas aeruginosa*	1	2.0
*Yeasts*	1	2.0

**Fig. 2:**
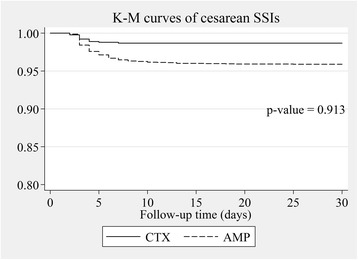
Adjusted overall cesarean SSIs survival by type of antibiotic prophylaxis. *P*-value was obtained from Cox’s survival regression analysis. AMP ampicillin group, CTX ceftriaxone group, K-M Kaplan-Meier, SSIs surgical site infections

**Fig. 3:**
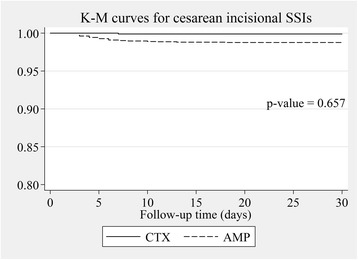
Adjusted cesarean incisional SSIs survival by type of antibiotic prophylaxis. *P*-value was obtained from Cox’s survival regression analysis. AMP ampicillin group, CTX ceftriaxone group, K-M Kaplan-Meier, SSIs surgical site infections

**Fig. 4:**
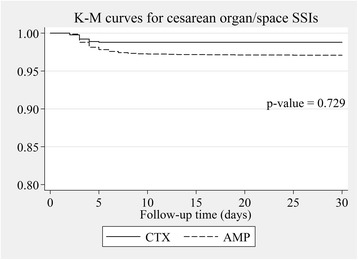
Adjusted cesarean organ/space SSIs survival by type of antibiotic prophylaxis. *P*-value was obtained from Cox’s survival regression analysis. AMP ampicillin group, CTX ceftriaxone group, K-M Kaplan-Meier, SSIs surgical site infections

After adjusting for propensity scores and other confounding factors included age, being referred, education level, body weight, being an ethnic minority, anemia, pelvic examination more than 4 before cesarean section, being preterm, wound class 3 or more, presence of foul-smelling amniotic fluid, longer than 55-min operative time, emergency operation, ASA-score of 3 or more, being primigravida, estimated blood loss, skin incision line and addition procedures; rate ratios with 95% confidence interval of total cesarean SSIs, incisional SSIs and organ/space SSIs in the ceftriaxone group compared with the ampicillin group were 1.10; 95% CI 0.58–2.08, 0.23; 95% CI 0.03–1.78 and 1.62; 95% CI 0.83–3.18, respectively (Table 4). The Kaplan-Meier survival curves of overall cesarean SSIs, incisional SSIs, and organ/space SSIs with *p*-values between the ceftriaxone and ampicillin groups are demonstrated in Figs. 2, 3 and 4. The Kaplan-Meier survival curves stratified by potential risk factors of cesarean SSIs are provided in the Additional file 1: Appendix.

**Table 4:** Risk ratio of cesarean SSIs comparing ceftriaxone to ampicillin groups after multilevel mixed-effects Poisson regression

Outcome	RR	95% Confidence interval	*p*-value
Total cesarean SSIs	1.10	0.58–2.08	0.765
Cesarean incisional SSIs	0.23	0.03–1.78	0.158
Cesarean organ/space SSIs	1.62	0.83–3.18	0.160

*RR* Risk ratio, *SSIs* Surgical site infections

## Discussion

Our study revealed a comparable efficacy of ampicillin and ceftriaxone, newer generation and broader antibacterial spectra-antibiotics, in preventing cesarean SSIs. In the antibiotic-resistant microorganism era, this is useful for promoting rational antibiotic use programs to prescribe more specific and narrower antibacterial spectra. As a result, it can prevent emerging multidrug resistant organisms after selective pressure from broad spectrum antibiotics overuse [21–24], a crisis that has been widely reported and tends to increase worldwide [25]. Ampicillin is also cheaper and may fit limited-resource settings.

The ideal study to compare the efficacy among antibiotic prophylaxis regimens is a randomized control design where all confounders are equally distributed on all comparison groups [26]. However, such a study design cannot be conducted in many circumstances in almost all routine clinical practices. In real practice, antibiotic prophylaxis is assigned for each patient based on beneficence and non-maleficence, under individual circumstances or indication and non-randomly. The observational study on assigned antibiotic prophylaxis to patients in the real context as in this study is optional and more feasible [27]. However, the confounders by indication among comparison groups in the observational study were inevitable [28–30]. In our study, demographic data differed between the two comparison groups. Confounding biases from an imbalance of baseline demographic data, acting as prognostic factors, should be controlled by adjusting in the model of regression [28]. We used propensity score to estimate probability of prescribing ceftriaxone for cesarean SSIs prophylaxis from abovementioned confounders by indication.

Propensity score or balancing score in other words, represented a scalar quantity that is calculated from a set of confounders by indication and contra-indication. It represents the conditional probability of receiving a particular treatment in the context and is used to control the effect of such confounders by adjusting it in the model of multivariable regression [31, 32].

The estimators for comparing of antibiotic prophylaxis groups concerning cesarean SSIs prevention in this study became non-different after confounders and propensity score was adjusted in the regression model (Table 4) and met the same direction as related well-designed studies [13].

The half-life of antibiotics used for SSIs prophylaxis is another important issue of concern. Redosing of antibiotic prophylaxis is needed when operative time is longer than two half-lives of the antibiotic used to ensure that the tissue level of the antibiotic is above the minimum inhibitory concentration of common pathogens of SSIs for that operation [9]. The half-lives of ampicillin and ceftriaxone are 1.7 ± 0.3 h [33, 34] and 6.3–6.9 h [35], respectively. Compared with the 75th percentile cesarean operation time reported by the National Nosocomial Infections Surveillance (NNIS) and in Thailand, including our setting, it required less than 60 min [16, 36, 37]. Therefore, the redosing of ampicillin and ceftriaxone in this situation may not be required. Additionally however, either two doses of ampicillin or a single dose of ceftriaxone was given within the first 24 h after cesarean section in the study setting. The use of a single dose preoperative antibiotic prophylaxis for cesarean delivery is a major challenge in this setting. In addition, the choice of antibiotic prophylaxis should be selected in accordance with standard guidelines [9, 12].

The strength of this study was the large study size that was sufficient to detect differences among groups. Post-hoc power analyses have shown that the power of this study for overall cesarean SSIs, cesarean incisional and organ/space SSIs was estimated to be 99.78, 96.18, and 89.01%, respectively. In addition, our observational design comprised a mixed-population that could represent real-world circumstances, so the results could be more generalized or applied. The propensity score technique with multilevel analysis and advanced statistical methods was used in data analysis to estimate the efficacy of both regimens. The results from our study were comparable to one related meta-analysis of randomized controlled trials [32]. This may be useful for periodic monitoring of the efficacy without conducting an experimental study. However, some limitations were revealed in this study. Propensity scores can incorporate only known confounding by indicated factors. They cannot adjust the effect of the unknown confounders or covariates that have not been collected for analysis [32, 38, 39]. Another limitation is its generalizability because the study was conducted in a single setting and all patients received either 3 doses of ampicillin or 2 doses of ceftriaxone in accordance with standing order protocol. The findings from our study might not be generalizable to other settings that prescribed the differences in dose and choice of antibiotic prophylaxis; however, it could be applied to similar settings. Pooled data from other sites should be studied in advance.

## Conclusions

The efficacy of ampicillin and ceftriaxone to prevent cesarean SSIs did not differ indicating that ampicillin may be used as an antibiotic prophylaxis in cesarean sections. Propensity scores can be used in data analysis of an observational study to compare the efficacy of antibiotic prophylaxis regimens and repeat performance as a monitoring measure.

## Additional file

Additional file 1: Appendix (DOCX 95 kb)

## Abbreviations

ASA: American Society of Anesthesiology
CDC: Centers for Disease Control and Prevention
dL: Decilitre
EBL: Estimated blood loss
G: gram
GA: Gestational age
Hb: Hemoglobin
HIV: Human immunodeficiency virus
Hr: hours
IQR: Interquartile range
Kg: Kilogram
Min: Minute
mL: Millilitre
MSAF: Meconium stained amniotic fluid
NNIS: National Nosocomial Infections Surveillance
PV: Pelvic examinations
ROM: Rupture of the membranes
RR: Rate ratios
SD: Standard deviation
SSIs: Surgical site infections
SSIs: Surgical site infections
TR: tubal resection
UTI: Urinary tract infection
Yr: Years

## References

[1] Osterman MJ, Martin JA (2014). Trends in low-risk cesarean delivery in the United States, 1990-2013. Natl Vital Stat Rep.

[2] Festin MR, Laopaiboon M, Pattanittum P, Ewens MR, Henderson-Smart DJ, Crowther CA (2009). Caesarean section in four south east Asian countries: reasons for, rates, associated care practices and health outcomes. BMC Pregnancy Childbirth.

[3] Edwards JR, Peterson KD, Mu Y, Banerjee S, Allen-Bridson K, Morrell G (2009). National Healthcare Safety Network (NHSN) report: data summary for 2006 through 2008, issued December 2009. Am J Infect Control.

[4] Kasatpibal N, Jamulitrat S, Chongsuvivatwong V (2005). Standardized incidence rates of surgical site infection: a multicenter study in Thailand. Am J Infect Control.

[5] Allegranzi B, Zayed B, Bischoff P, Kubilay NZ, de Jonge S, de Vries F, et al. New WHO recommendations on intraoperative and postoperative measures for surgical site infection prevention: an evidence-based global perspective. Lancet Infect Dis. 2016;16(12):e288-e303. 10.1016/S1473-3099(16)30402-9 27816414

[6] The American College of Obstetricians and Gynecologists. ACOG Committee Opinion No (2010). 465: antimicrobial prophylaxis for cesarean delivery: timing of administration. Obstet Gynecol.

[7] Berríos-Torres SI, Umscheid CA, Bratzler DW, et al. Centers for disease control and prevention guideline for the prevention of surgical site infection. JAMA Surg. 2017;152(8):784-91. 10.1001/jamasurg.2017.0904 28467526

[8] Smaill Fiona M, Grivell RM. Antibiotic prophylaxis versus no prophylaxis for preventing infection after cesarean section. Cochrane Database Syst Rev. 2014;10 10.1002/14651858.CD007482.pub3 PMC8078551 25350672

[9] Bratzler DW, Dellinger EP, Olsen KM, Perl TM, Auwaerter PG, Bolon MK (2013). Clinical practice guidelines for antimicrobial prophylaxis in surgery. Am J Health Syst Pharm.

[10] Kasatpibal N, Whitney JD, Dellinger EP, Nair BG, Pike KC (2017). Failure to Redose antibiotic prophylaxis in long surgery increases risk of surgical site infection. Surg Infect.

[11] Pai MP, Bearden DT (2007). Antimicrobial dosing considerations in obese adult patients. Pharmacotherapy.

[12] The American College of Obstetricians and Gynecologists. ACOG Practice Bulletin No (2011). 120: use of prophylactic antibiotics in labor and delivery. Obstet Gynecol.

[13] Gyte GM, Dou L, Vazquez JC. Different classes of antibiotics given to women routinely for preventing infection at caesarean section. Cochrane Database Syst Rev. 2014;(11):1-191. 10.1002/14651858.CD008726.pub2 PMC7173707 25402227

[14] Mangram AJ, Horan TC, Pearson ML, Silver LC, Jarvis WR (1999). Guideline for prevention of surgical site infection, 1999. Hospital infection control practices advisory committee. Infect Control Hosp Epidemiol.

[15] Horan TC, Andrus M, Dudeck MA (2008). CDC/NHSN surveillance definition of health care-associated infection and criteria for specific types of infections in the acute care setting. Am J Infect Control.

[16] Assawapalanggool S, Kasatpibal N, Sirichotiyakul S, Arora R, Suntornlimsiri W (2016). Risk factors for cesarean surgical site infections at a Thai-Myanmar border hospital. Am J Infect Control.

[17] Rubin DB, Thomas N (2000). Combining propensity score matching with additional adjustments for prognostic covariates. J Amer Statistical Assoc.

[18] Austin PC (2011). An introduction to propensity score methods for reducing the effects of confounding in observational studies. Multivariate Behav Res.

[19] Nguyen TL, Collins GS, Spence J, Daures JP, Devereaux PJ, Landais P (2017). Double-adjustment in propensity score matching analysis: choosing a threshold for considering residual imbalance. BMC Med Res Methodol.

[20] Grobbee DE, Hoes AW (2015). Clinical epidemiology: principles, methods, and applications for clinical research.

[21] Pouladfar G, Jafarpour Z, Hosseini SA, Janghorban P, Roozbeh J (2015). Antibiotic selective pressure and development of bacterial resistance detected in bacteriuria following kidney transplantation. Transplant Proc.

[22] Alonso A, Campanario E, Martinez JL (1999). Emergence of multidrug-resistant mutants is increased under antibiotic selective pressure in Pseudomonas Aeruginosa. Microbiology.

[23] Kolar M, Urbanek K, Latal T (2001). Antibiotic selective pressure and development of bacterial resistance. Int J Antimicrob Agents.

[24] Dinubile MJ, Friedland I, Chan CY, Motyl MR, Giezek H, Shivaprakash M (2005). Bowel colonization with resistant gram-negative bacilli after antimicrobial therapy of intra-abdominal infections: observations from two randomized comparative clinical trials of ertapenem therapy. Eur J Clin Microbiol Infect Dis.

[25] World Health Organization (2014). Antimicrobial resistance: global report on surveillance.

[26] Sibbald B, Roland M (1998). Understanding controlled trials. Why are randomised controlled trials important?. BMJ.

[27] Fletcher RH, Fletcher SW, Fletcher RH, Fletcher SW (2005). Treatment. Clinical epidemiology: the essentials.

[28] Kramer MS, Kramer MS (1988). Analytic bias. Clinical epidemiology and biostatistics.

[29] Hullsiek KH, Louis TA (2002). Propensity score modeling strategies for the causal analysis of observational data. Biostatistics.

[30] Miettinen OS (1983). The need for randomization in the study of intended effects. Stat Med.

[31] D’Agostino RB (1998). Propensity score methods for bias reduction in the comparison of a treatment to a non-randomized control group. Stat Med.

[32] Austin PC (2014). The use of propensity score methods with survival or time-to-event outcomes: reporting measures of effect similar to those used in randomized experiments. Stat Med.

[33] Sjovall J, Alvan G, Huitfeldt B (1986). Intra- and inter-individual variation in pharmacokinetics of intravenously infused amoxycillin and ampicillin to elderly volunteers. Br J Clin Pharmacol.

[34] Ehrnebo M, Nilsson SO, Boreus LO (1979). Pharmacokinetics of ampicillin and its prodrugs bacampicillin and pivampicillin in man. J Pharmacokinet Biopharm.

[35] Yuk JH, Nightingale CH, Quintiliani R (1989). Clinical pharmacokinetics of ceftriaxone. Clin Pharmacokinet.

[36] Kasatpibal N, Norgaard M, Jamulitrat S (2009). Improving surveillance system and surgical site infection rates through a network: a pilot study from Thailand. Clin Epidemiol.

[37] Centers for Disease Control and Prevention (2004). National Nosocomial Infections Surveillance (NNIS) system report, data summary from January 1992 through June 2004, issued October 2004. Am J Infect Control.

[38] Shah BR, Laupacis A, Hux JE, Austin PC (2005). Propensity score methods gave similar results to traditional regression modeling in observational studies: a systematic review. J Clin Epidemiol.

[39] Trojano M, Pellegrini F, Paolicelli D, Fuiani A, Di Renzo V (2009). Observational studies: propensity score analysis of non-randomized data. Int MS J.

