# Supplementary material for: The efficacy of ampicillin compared with ceftriaxone on preventing cesarean surgical site infections: an observational prospective cohort study
Source: Antimicrob Resist Infect Control. 2018 Jan 22;7:13. doi: 10.1186/s13756-018-0304-6 (PMC5778626; doi:10.1186/s13756-018-0304-6)
Supplement: Additional file 1: — Appendix (DOCX 95 kb) [file 13756_2018_304_MOESM1_ESM.docx]

**Appendix**

|  |  |  |
| --- | --- | --- |
|  |  |  |
|  |  |  |
|  |  |  |
| **Fig. 5** Kaplan-Meier curves of cesarean incisional SSIs stratified by potential risk factors of SSIs  P-values were obtained from Cox’s survival regression analysis.  AF amniotic fluid, AMP ampicillin, ASA American Society of Anesthesiology, CTX ceftriaxone, exam examination, min minutes, OPT operative time, PV pelvic examinations, SSIs surgical site infections | | |

|  |  |  |
| --- | --- | --- |
|  |  |  |
|  |  |  |
|  |  |  |
| **Fig. 6** Kaplan-Meier curves of cesarean organ/space SSIs stratified by potential risk factors of SSIs  P-values were obtained from Cox’s survival regression analysis.  AF amniotic fluid, AMP ampicillin, ASA American Society of Anesthesiology, CTX ceftriaxone, exam examination, min minutes, OPT operative time, PV pelvic examinations, SSIs surgical site infections | | |
